# Targeted Radiotherapy in Primary Cutaneous Lymphomas: Precision, Efficacy, and Evolving Strategies

**DOI:** 10.3390/cancers17172722

**Published:** 2025-08-22

**Authors:** Piotr Sobolewski, Mateusz Koper, Piotr Ciechanowicz, Irena Walecka

**Affiliations:** Dermatology Clinic, National Institute of Medicine of the Ministry of Interior and Administration, 02-507 Warsaw, Poland; piotr.sobolewski@pimmswia.gov.pl (P.S.); piotr.ciechanowicz@pimmswia.gov.pl (P.C.); irena.walecka@pimmswia.gov.pl (I.W.)

**Keywords:** primary cutaneous lymphomas, mycosis fungoides, radiotherapy

## Abstract

Primary cutaneous lymphomas (PCLs), including cutaneous T-cell lymphomas (CTCL) and primary cutaneous B-cell lymphomas (PCBCL), are a diverse group of non-Hodgkin lymphomas that primarily affect the skin. Radiotherapy (RT) plays a pivotal role in the treatment of these lymphomas, particularly for localized disease, due to its ability to deliver precise, skin-directed treatment. Mycosis fungoides (MF) and Sézary syndrome (SS), the most common subtypes of CTCL, often require skin-directed therapies such as electron beam therapy and superficial brachytherapy to manage localized lesions. Electron beam therapy, including total skin electron beam therapy (TSEBT), has been utilized for decades, offering high response rates but with the risk of cumulative skin toxicity. Recently, low-dose radiotherapy (LDRT) has gained attention as an effective alternative that reduces toxicity while maintaining durable responses. Superficial brachytherapy is another modality that delivers radiation through custom molds, allowing for homogeneous dosing over complex anatomical areas like the face. Both teleradiotherapy and brachytherapy have demonstrated high complete response rates, with low recurrence rates observed when higher doses are used. In the context of primary cutaneous B-cell lymphomas, such as primary cutaneous marginal zone lymphoma (PCMZL) and primary cutaneous follicle center lymphoma (PCFCL), radiotherapy also offers excellent local control, particularly for indolent subtypes. However, more aggressive subtypes, such as diffuse large B-cell lymphoma, leg type (PCDLBCL-LT), may require systemic therapies in addition to radiation. Overall, teleradiotherapy and brachytherapy are essential components of the therapeutic arsenal for primary cutaneous lymphomas, offering effective disease control with manageable toxicity, while ongoing research focuses on optimizing treatment strategies and exploring novel combinations with systemic therapies.

## Simple Summary

Primary cutaneous lymphomas (PCLs) are rare non-Hodgkin lymphomas affecting the skin, mainly categorized into cutaneous T-cell lymphomas (CTCL) and cutaneous B-cell lymphomas (CBCL). Radiotherapy (RT), including external beam therapy and brachytherapy (BT), plays a key role in treating localized disease, offering high response rates with manageable toxicity. Mycosis fungoides (MF) and Sézary syndrome, the main CTCL subtypes, benefit from treatments like total skin electron beam therapy (TSEBT) and low-dose RT, which reduce toxicity while maintaining effectiveness. CBCLs, such as primary cutaneous marginal zone lymphoma and follicle center lymphoma, respond well to RT, whereas aggressive types like leg-type diffuse large B-cell lymphoma may require systemic therapies. Brachytherapy, particularly high-dose rate (HDR) techniques using custom molds, has emerged as an effective option for treating superficial or anatomically complex lesions. RT provides excellent local control, especially in early-stage disease. However, skin toxicity, including erythema and desquamation, is common and requires supportive care. Future research aims to refine these therapies, optimize dosing, and combine them with systemic treatments to improve long-term outcomes and patient quality of life.

## 1. Introduction

**Figure 1:**
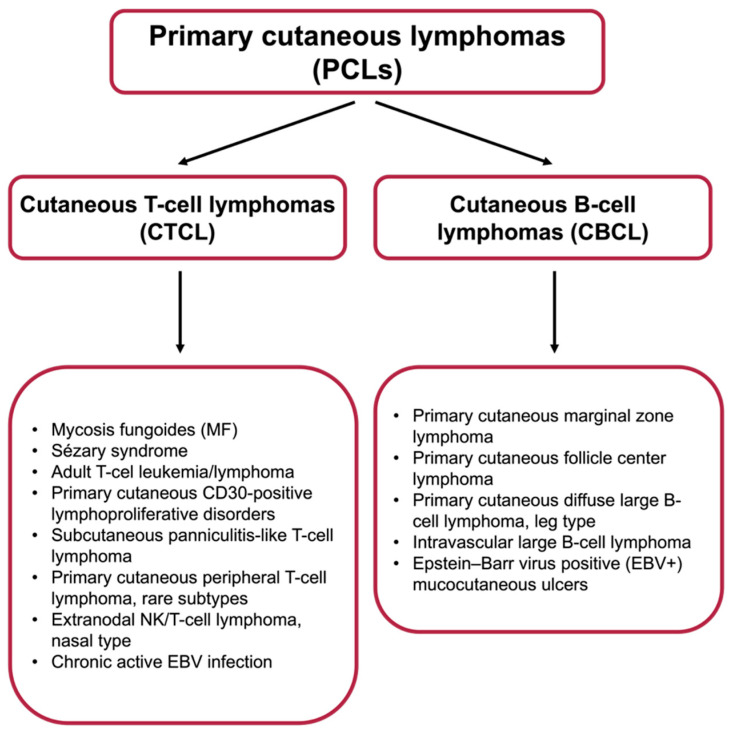
Classification of PCLs based on the 2018 WHO–EORTC classification update [2].

Primary cutaneous lymphomas (PCLs) represent a rare and heterogeneous group of non-Hodgkin lymphomas, primarily involving the skin. These lymphomas can be further classified into two main subtypes: primary cutaneous B-cell lymphomas (PCBCLs) and primary cutaneous T-cell lymphomas (PCTCLs), with each displaying unique clinical, histopathological, and prognostic features [1]. Understanding the nature of these lymphomas and their optimal treatment strategies is critical in delivering effective, personalized patient care. A detailed classification of PCLs is presented in Figure 1.

PCLs generally exhibit high sensitivity to radiotherapy, making it a particularly effective treatment option. Many PCL subtypes, such as mycosis fungoides and certain other T-cell and B-cell cutaneous lymphomas, respond well to localized radiation, often achieving significant symptom relief and durable remission in treated areas [3,4]. Despite this, the potential of radiotherapy in managing PCLs remains underutilized. Expanding the use of advanced radiotherapy techniques could enhance treatment outcomes, particularly for patients with limited disease or those unresponsive to other therapies [5]. This highlights an untapped potential for radiotherapy to play a larger role in both palliative and curative strategies for PCLs.

PCBCLs account for approximately 25% of all cutaneous lymphomas [6]. These lymphomas arise from B-lymphocytes and are divided into three major subtypes according to the World Health Organization (WHO) 2017 classification: primary cutaneous marginal zone lymphoma, primary cutaneous follicle center lymphoma, and primary cutaneous diffuse large B-cell lymphoma, leg type. Additional rare variants, such as intravascular large B-cell lymphoma, are also recognized. In September 2018, the WHO-European Organization for Research and Treatment of Cancer (WHO-EORTC) added Epstein–Barr virus-positive (EBV+) mucocutaneous ulcers as a distinct entity under the umbrella of cutaneous B-cell lymphomas [2]. The incidence of PCBCLs has been increasing, with current estimates around four per million persons, predominantly affecting older adults, particularly males [7].

Diagnosis of PCBCLs is based on a combination of clinical, histopathological, and immunohistochemical analyses, often supplemented with dermoscopic examination [8]. For a definitive diagnosis, histological examination of skin biopsies—either excisional or punch—is essential, along with comprehensive staging procedures [9].

Management of PCBCLs varies depending on the subtype and disease extent. PCMZL and PCFCL are generally considered indolent lymphomas with a favorable prognosis, boasting a 5-year disease-specific survival rate exceeding 95%. In contrast, PCDLBCL, LT is more aggressive, with a 5-year survival rate below 60% [10]. Treatment strategies range from observation to localized and systemic therapies, often involving a multidisciplinary team consisting of dermatologists, pathologists, haemato-oncologists, and radiation oncologists. Radiotherapy plays a pivotal role in managing PCBCLs, especially in cases with solitary or localized lesions, offering high response rates and long-term control with minimal toxicity [11].

In parallel, PCTCLs account for approximately two-thirds of all cutaneous lymphomas. The most common subtype, mycosis fungoides (MF), and its more aggressive leukemic variant, Sézary syndrome (SS), originate from monoclonal proliferation of T-lymphocytes, typically CD4+ T-cells with a predilection for the skin [12]. Early-stage mycosis fungoides tends to follow an indolent course in the majority of patients. However, around 20% of these patients may develop aggressive, life-threatening disease [13]. Most therapies are aimed at disease control rather than cure, with frequent recurrences observed, particularly in early-stage mycosis fungoides. Radiotherapy has emerged as a highly effective treatment modality for CTCL, particularly in localized skin lesions [14]. Superficial radiation therapies, such as electron beam therapy and brachytherapy, are frequently employed, with electron beam therapy offering the advantage of limited penetration beyond the skin, thus sparing deeper tissues [15]. However, it has limitations when treating areas with complex topography. Brachytherapy, which uses surface molds and flexible catheters to deliver radiation directly to the affected area, can achieve more homogeneous dosing over irregular surfaces, such as the face, while minimizing collateral damage [16]. In this article, we analyze the treatment of PCLs using teleradiotherapy and brachytherapy, focusing on their effectiveness in managing localized skin disease.

## 2. Radiotherapy in Skin Cancers Treatment

Radiotherapy is an established and highly effective treatment modality in skin cancers, particularly for patients who are not surgical candidates or in cases where surgery may result in significant cosmetic or functional impairment [17]. It is commonly employed in basal cell carcinoma, squamous cell carcinoma, and primary cutaneous lymphomas, with excellent local control rates and favorable cosmetic outcomes [18,19]. Different techniques—including superficial X-rays, electron beam therapy, and brachytherapy—allow for precise tailoring of radiation delivery to the tumor’s size, depth, and anatomical location, thereby minimizing toxicity to surrounding healthy tissue [20]. Hypofractionated regimens are frequently used in elderly or frail patients, offering convenience while maintaining high efficacy [21]. RT is also indicated in advanced or recurrent disease, often as part of a multidisciplinary approach, either for definitive treatment, palliation, or in combination with systemic therapies such as immunotherapy. Importantly, beyond its cytotoxic effect, RT in skin cancers has been increasingly recognized for its immunomodulatory potential, capable of enhancing antitumor immune responses and contributing to systemic disease control [22]. Comparison of radiotherapy modalities is presented in Table 1.

RT in skin cancers exerts its antitumor activity not only through direct cytotoxic effects but also by reshaping the tumor microenvironment (TME) in a manner that promotes immune activation [23]. Low-dose RT has been shown to preferentially induce proinflammatory M1-like macrophage polarization, enrich natural killer (NK) cell infiltration, and enhance trafficking of immune cells into cutaneous lesions, thereby amplifying local immune surveillance [24]. At the molecular level, RT-induced double-stranded DNA breaks lead to cytosolic DNA accumulation, which activates cyclic GMP-AMP synthase (cGAS), triggering interferon release and further immune stimulation [25,26]. However, higher doses (>12–18 Gy) can induce the DNA exonuclease TREX1 (three-prime repair exonuclease 1), which degrades cytoplasmic DNA and thereby attenuates RT-induced immune responses [25,27]. RT also upregulates a spectrum of immunologically relevant molecules, including ligands, death receptors, neoantigens, chemokines, and proinflammatory cytokines such as IFN-γ, while stimulating CD8+ T cell proliferation [28,29,30].

Low-dose local radiotherapy represents a highly effective option for a wide range of primary cutaneous lymphomas, providing prompt symptom control and clearance of skin lesions with minimal toxicity and relatively low rates of in-field recurrence [19].

**Table 1:** Comparison of radiotherapy (RT) modalities in primary cutaneous lymphomas.

Modality	Indications	Pros	Cons	Outcomes	Ref.
**Brachytherapy (HDR/Surface)**	Superficial, small, or irregularly shaped lesions; sites requiring tissue preservation	Precise dose delivery; spares surrounding tissues; excellent cosmetic results	Limited availability of HDR units; requires specialized expertise	Excellent local control; high patient satisfaction with cosmetic outcome	[31,32,33]
**Total Skin Electron Beam Therapy (TSEBT)**	Widespread cutaneous involvement (e.g., advanced MF/SS)	Covers entire skin surface; effective palliation; rapid symptom relief	Technically complex; requires specialized equipment; relapse common	High response rates, but frequent relapses; best for palliation or induction therapy	[15,34]
**Low-dose RT (localized or TSEBT)**	Elderly/frail patients; relapsed disease; palliative settings	Low toxicity; convenient; can be repeated; immunomodulatory effects	Responses often short-lived; not curative as monotherapy	Good short-term control; useful bridge to systemic or combined therapy	[35,36]

### 2.1. Radiotherapy in the Treatment of Primary Cutaneous B-Cell Lymphomas

PCBCLs typically present as indolent, slow-growing lesions confined to the skin. Due to their localized nature and the efficacy of targeted treatment modalities, radiotherapy has become a cornerstone in the management of CBCLs. RT offers a highly precise and effective approach for controlling disease while minimizing systemic side effects, making it an ideal treatment option for many patients with CBCLs [7].

Radiotherapy plays a pivotal role in the treatment of CBCLs due to its ability to precisely target and eliminate malignant cells confined to the skin. Unlike systemic therapies, which may have broader impacts on the body, RT focuses specifically on the affected areas, delivering a localized and potent dose of radiation to the lymphoma lesions while sparing surrounding healthy tissue [37]. This targeted approach allows for excellent disease control, with minimal systemic toxicity.

Radiation therapy is often employed as a first-line treatment for localized disease, especially in cases of indolent subtypes such as PCFCL and PCMZL, which typically have a good prognosis [38,39]. For instance, studies have demonstrated that localized radiation can lead to high rates of complete response in these indolent forms, with minimal adverse effects [38,39]. In contrast, PCDLBCL, particularly the leg type, is associated with a more aggressive clinical course and poorer prognosis, often requiring a combination of systemic therapies alongside radiotherapy [40,41]. The modalities of teleradiotherapy utilized in the treatment of PCBCL include conventional external beam radiation therapy (EBRT) and more advanced techniques such as intensity-modulated radiation therapy (IMRT) and electron beam therapy (EBT). IMRT allows for precise targeting of tumor tissues while sparing surrounding healthy skin, which is particularly beneficial in treating lesions located on sensitive areas of the body [1]. EBT is also well-established for treating skin lesions due to its ability to deliver high doses of radiation to superficial tumors with minimal penetration into deeper tissues [1,42]. In cases where PCBCL presents with extensive skin involvement or is associated with systemic symptoms, the treatment approach may shift towards more aggressive regimens, including the use of systemic chemotherapy combined with involved-site radiation therapy (ISRT) [40,41]. The R-CHOP regimen (rituximab, cyclophosphamide, doxorubicin, vincristine, and prednisone) is commonly employed for PCDLBCL, with radiotherapy serving as an adjunct to enhance local control and reduce the risk of recurrence [40,41]. Moreover, the integration of immunotherapy, particularly the use of monoclonal antibodies like rituximab, has shown promise in improving outcomes for patients with PCBCL, especially in those with more aggressive disease [39,43]. The combination of rituximab with teleradiotherapy has been associated with improved response rates and prolonged survival in patients with PCDLBCL [39,43]. The management of PCBCL also necessitates careful monitoring for potential complications associated with teleradiotherapy. Skin reactions, including erythema and desquamation, are common but typically resolve with appropriate supportive care [1,42]. Long-term follow-up is essential to assess for late effects of radiation, including the risk of secondary malignancies, which, although rare, can occur following radiation treatment for PCBCL [1,41].

Lesions in primary cutaneous B-cell lymphomas are typically categorized by their size and spread. In the case of solitary lesions, classified as T1 (T1a < 5 cm in diameter; T1b ≥ 5 cm in diameter), radiotherapy is often delivered using “involved lesion RT,” which focuses on treating the lymphoma itself while ensuring sufficient margins to account for any microscopic disease that may not be visible. These margins are crucial to prevent recurrence by treating areas of potential microscopic spread beyond the clinically apparent lesion [1]. The size of the margins around the lesion is an important consideration in delivering effective radiotherapy. Margins beyond the clinically evident erythema or induration of the lesion are needed to ensure that any microscopic disease surrounding the lesion is adequately treated. The appropriate size of the margin can vary based on factors such as the size of the lesion and the anatomical location of the tumor. Recommended margin sizes in the literature typically range from 0.5 cm to 5.0 cm [4,5,44,45,46], with the European Organization for Research and Treatment of Cancer and the International Society for Cutaneous Lymphomas (ISCL) recommending a margin of 1.0 to 1.5 cm for both primary cutaneous follicle center lymphoma and primary cutaneous marginal zone lymphoma [3]. The International Lymphoma Radiation Oncology Group (ILROG) similarly endorses these recommendations, emphasizing the importance of consistent and adequate margins for disease control. In addition to lateral margins, it is important to consider the depth of the lesion when planning RT. The thickness of the lesion must be carefully assessed to ensure that the radiation dose adequately covers not only the surface of the lesion but also any underlying tissue that may harbor microscopic disease. Typically, the depth margin mirrors the lateral margin, though adjustments may be necessary based on the lesion’s location and the characteristics of the soft tissue beneath it. In areas where intact bone or fascia lies beneath the lesion, it is often unnecessary to extend the radiation coverage deeper, as these structures are less likely to harbor lymphoma cells [1].

In CBCLs, including PCFCL and PCMZL, radiotherapy is highly effective, with curative doses generally delivered in the range of 20–40 Gy. While individual guideline recommendations differ slightly, major international bodies such as EORTC/ISCL, National Comprehensive Cancer Network (NCCN), and ILROG all converge on this moderate-dose approach as the optimal balance between disease control and treatment tolerability. These recommendations are supported by both clinical trial data in indolent lymphomas and accumulated real-world experience in cutaneous disease, demonstrating consistently high local control rates, durable remissions in many cases, and low toxicity [3,4,5,44,45,47].

While these doses are highly effective for achieving remission in most patients, it is important to note that lower-dose regimens may also be effective for select patients. For example, palliative doses of teleradiotherapy, such as 2 Gy administered over two sessions, have been shown to achieve complete remission in 72% of cases. However, approximately 30% of these lesions may require retreatment within a median of 6.3 months, suggesting that while palliative doses can provide temporary relief, they may not offer long-term disease control [46]. On the other hand, the concept of using low-dose radiotherapy with 4 Gy demonstrated a similar local control rate (LCR) compared to higher doses, but it was associated with lower response rates and reduced acute toxicity. Due to the significantly lower response rates observed with LDRT, it may not be recommended as the standard treatment approach for PCBCL [11].

In summary, teleradiotherapy is a cornerstone in the treatment of primary cutaneous B-cell lymphomas, particularly for localized disease. The choice of radiotherapy technique, whether conventional EBRT, IMRT, or EBT, depends on the specific clinical scenario, including the lymphoma subtype and the extent of skin involvement. As the field of oncology continues to evolve, ongoing research into the optimal integration of radiotherapy with systemic therapies will be crucial in enhancing treatment outcomes for patients with PCBCL.

### 2.2. Radiotherapy in the Treatment of Primary Cutaneous T-Cell Lymphomas

Radiotherapy is a critical component in the management of cutaneous T-cell lymphomas, particularly mycosis fungoides (MF), which is the most common subtype of CTCL. This malignancy is characterized by the proliferation of malignant T-lymphocytes primarily in the skin, leading to a variety of clinical presentations, including patches, plaques, and tumors. The role of radiotherapy in CTCL, especially in early-stage MF, has been extensively studied and is supported by clinical evidence demonstrating its efficacy in achieving local control of the disease and improving patient outcomes [48].

Mycosis fungoides predominantly affects the skin but may progress to involve lymph nodes, blood, and other organs in advanced stages. The treatment of MF is often based on its clinical stage, and radiotherapy plays a crucial role in managing the disease, particularly in localized and advanced forms. Prognostic factors also play a crucial role in determining treatment strategies for MF. Studies have indicated that factors such as age, race, and the extent of skin involvement can significantly influence outcomes [49,50]. One of the most effective treatment options for mycosis fungoides is total skin electron beam therapy, a form of teleradiotherapy that targets the entire skin surface. Over time, TSEBT has become a cornerstone for skin-directed treatments in MF, particularly because it can address widespread cutaneous involvement, sparing deeper tissues from damage. Historically, TSEBT doses were gradually increased from 8 Gy to 36 Gy, with studies showing that higher doses led to better complete response (CR) rates while remaining tolerable to patients [15,51]. However, TSEBT is dose-dependent, and side effects become more common with higher doses. Common side effects of TSEBT include erythema and dry desquamation, conditions where the skin becomes red and peels off. At a dose of 36 Gy, patients may experience more severe intermediate and late side effects, such as temporary loss of sweating (anhidrosis) and loss of fingernails or toenails. Hair loss, or alopecia, may also occur and can become irreversible at doses exceeding 25 Gy [52]. Despite these side effects, many patients experience a recurrence of the disease even after receiving 36 Gy. This presents a challenge in treatment because repeated high-dose TSEBT carries risks of severe skin atrophy and xerosis (dryness), limiting the possibility of administering more than two courses of 36 Gy TSEBT [53,54].

#### 2.2.1. Localized Teleradiotherapy for MF Lesions

The staging of MF/SS relies on the tumor–node–metastasis (TNM) classification system, which was initially developed in 1979 [55]. In 2007, this system was revised and expanded to incorporate blood involvement, resulting in the TNMB classification [56].

For patients presenting with T1 MF lesions (patches or plaques covering less than 10% of the total skin surface), localized radiotherapy alone may be sufficient. This treatment can also be applied to patients with clustered T2 lesions (generalized patch/plaque covering more than 10% of the skin) or T3 lesions (tumors of at least 1 cm in diameter). Total skin electron beam therapy is particularly indicated for patients with diffuse T2 lesions or T4 skin lesions, which involve erythema covering more than 80% of the body surface area [57]. O’Malley and colleagues demonstrated that radiotherapy can effectively eradicate malignant T-cells, leading to improved survival outcomes in patients with early-stage disease. This is particularly important for high-risk patients, where skin-directed therapies such as radiotherapy can significantly improve overall survival rates. Low-dose radiation regimens of more than 7 Gy are gaining popularity in clinical practice due to their favorable safety profile and effectiveness in managing MF. In a clinical trial, patients with mycosis fungoides were treated with low-dose radiotherapy at 8 Gy. Of the 16 lesions treated with LDRT, 5 achieved complete eradication of the malignant clone, while 11 lesions experienced a reduction of more than 90%. Notably, there were no recurrences in the lesions treated with LDRT, underscoring its efficacy [16].

#### 2.2.2. Clinical Stage IA Mycosis Fungoides: Teleradiotherapy as a Cure

Patients diagnosed with early-stage MF, particularly clinical stage IA, typically have patches or plaques covering less than 10% of their body surface. These patients do not exhibit significant involvement of the blood, lymph nodes, or internal organs, making them suitable candidates for localized treatments. A subset of patients within this group, known as those with “minimal” stage IA MF (characterized by having one to three localized lesions), have a particularly favorable prognosis. In fact, these patients can sometimes achieve long-term remission or even potential “cure” with localized radiotherapy alone [1]. Wilson et al. conducted a study evaluating 21 patients with minimal disease, 13 of whom had unilesional MF. These patients were treated with localized radiotherapy, and the results were highly promising. The complete response rate to localized RT was 97%, with disease-free survival (DFS) at 5 and 10 years reported at 75% and 64%, respectively. Importantly, patients who received doses of at least 20 Gy had a 91% DFS rate, highlighting the efficacy of adequate dosing in achieving long-term control of the disease [58]. Another study by Micaily and colleagues reported similarly encouraging outcomes in 18 patients with unilesional stage IA MF. Most of these patients received 30.6 Gy of radiotherapy, achieving a remarkable 100% CR rate. Furthermore, the relapse-free survival (RFS) and overall survival at 10 years were 86% and 100%, respectively, demonstrating the potential for radiotherapy to provide durable disease control in patients with localized MF. Although two patients did experience relapses, these were confined to distant skin sites and were effectively treated with topical nitrogen mustard [59]. Piccinno et al. also evaluated 15 patients with minimal stage MF treated with a median dose of 22 Gy. Their study found that 95% of patients achieved complete remission of the treated lesions, while 5% experienced partial remission. The relapse-free rate at 5 and 10 years was 51%, underscoring the need for ongoing monitoring and management even in patients who initially respond well to treatment [60].

#### 2.2.3. Teleradiotherapy for Advanced-Stage MF (IIB-III)

In more advanced cases of MF, where patients present with larger or thicker plaques and tumors (classified as stage IIB-III), localized radiotherapy or electron beam therapy is frequently employed. EBT targets the superficial layers of the skin, making it ideal for treating widespread skin involvement while sparing deeper tissues and organs. Total skin electron beam therapy is often used in advanced-stage MF or erythrodermic MF, a variant that involves the entire skin surface. Low-dose TSEBT, typically administered at 12 Gy, provides a safe and effective option for reducing the tumor burden in patients with stage IB to IIIA MF. Studies have shown that low-dose TSEBT can achieve response rates exceeding 88%, making it a viable alternative to the standard 36-Gy dose. Although the standard dose may offer higher initial complete response rates and longer durations of response, the lower dose is better tolerated and associated with fewer side effects. Furthermore, the transient nature of adverse events (AEs) at lower doses makes it a safer option for selected patients [61]. In one study, low-dose TSEBT administered at 12 Gy over 8 fractions in a period of 2 weeks resulted in an 18% complete response rate, a 69% partial response rate, and stable disease in 8% of patients. Only 5% of patients experienced progression during treatment [62]. Another study evaluating low-dose TSEBT at 10 Gy in 10 fractions demonstrated a 95% overall response rate, with a complete or near-complete response (less than 1% skin involvement) in 57% of patients. This approach offers the possibility of reirradiation in the event of relapse or disease progression, with lower toxicity compared to standard-dose TSEBT [36].

#### 2.2.4. Palliative Radiotherapy for Late-Stage MF

In patients with late-stage MF or Sézary syndrome, where skin tumors are more extensive or refractory, radiotherapy is often used palliatively to control symptoms such as ulceration, pain, and itching. For example, hemibody radiotherapy has been used in cases where the disease has progressed significantly. One patient received 6 Gy in 2 Gy fractions, resulting in significant clinical relief [63]. High-dose rate (HDR) brachytherapy has also been explored as an alternative treatment for folliculotropic mycosis fungoides (FMF), particularly for lesions with complex topography. HDR brachytherapy provides effective target coverage while sparing normal tissue, resulting in fewer adverse effects and a shorter treatment course compared to electron beam radiotherapy [64].

In conclusion, radiotherapy remains a vital component of mycosis fungoides treatment, offering options for both localized disease control and palliative care in advanced stages. As research continues, the development of safer, more effective radiotherapy techniques will likely improve the prognosis and quality of life for patients with MF.

#### 2.2.5. Brachytherapy in the Treatment of Primary Cutaneous Lymphomas

Brachytherapy (BT) has been a prominent form of radiotherapy for various cancers, and its use in treating primary cutaneous lymphomas has garnered attention for its precision, effectiveness, and relatively low toxicity compared to other methods. PCLs, such as cutaneous T-cell lymphoma and cutaneous B-cell lymphoma, are rare forms of non-Hodgkin lymphomas that manifest primarily on the skin without significant systemic involvement [33]. The treatment landscape for these conditions has evolved, with brachytherapy showing promise, particularly in cases where surgery or external beam radiation therapy may not be ideal. Brachytherapy involves placing a radioactive source close to or within the tumor, allowing high doses of radiation to be delivered to the tumor while sparing surrounding healthy tissue [65]. This method is particularly advantageous for treating superficial lesions, making it a fitting option for cutaneous lymphomas.

The use of HDR-BT in CBCLs is relatively new, with few reports documenting its application. One of the most significant contributions to the field was a case series by Chyrek et al., which provided valuable insights into HDR-BT’s effectiveness for CBCL. This study, conducted between 2011 and 2019, reported on seven patients with 12 histopathologically confirmed CBCL lesions. The majority of these lesions were categorized as T2a, with a few classified as T1a. HDR-BT was used as the first-line treatment for most cases, although four patients had previously undergone surgery, and one case involved adjuvant treatment after surgery. The median total dose administered was 36 Gy, delivered over 10 fractions, with treatment completed within an 11-day period. The results were highly encouraging, with a 100% local control rate observed over a mean follow-up period of 41 months. This level of control demonstrates HDR-BT’s effectiveness in treating CBCL, even in patients with recurrent disease. Although HDR-BT offers excellent local control, the treatment is not without its side effects. In the Chyrek et al. study, early toxicity was observed in the form of erythema (33%), patchy epidermal desquamation (25%), confluent epidermal desquamation (25%), and minor bleeding (17%). Late toxicity, though less severe, was characterized primarily by cosmetic concerns, including slight depigmentation in 59% of cases, small telangiectasia in 8%, massive telangiectasia in 25%, and one instance of small ulceration. Despite these side effects, the overall toxicity profile was manageable, with most side effects being mild and transient. The relatively low incidence of severe late toxicity makes HDR-BT a viable option for patients concerned about long-term cosmetic outcomes [33].

The use of brachytherapy in pediatric cutaneous lymphomas is rare, but a notable case by Linggonegoro et al. highlights its potential. In this instance, a six-year-old girl with primary cutaneous anaplastic large cell lymphoma (PC-ALCL) was treated successfully with HDR-BT. The lesion, which presented as a violaceous nodule on the forearm, was initially considered for surgical excision. However, due to concerns about scarring and joint contractures, brachytherapy was chosen. The patient received 20 Gy over eight daily fractions, and within six weeks, the lesion flattened, leaving only residual hyperpigmentation. After 21 months of follow-up, there was no evidence of recurrence, and the patient experienced only mild radiation dermatitis, which resolved on its own. This case underscores the benefits of HDR-BT in pediatric patients, particularly when surgery may result in undesirable cosmetic or functional outcomes. Brachytherapy’s precision and tissue-sparing properties make it a valuable option for treating pediatric cutaneous lymphomas, where long-term cosmetic and functional outcomes are of paramount importance [66].

Cutaneous T-cell lymphomas, particularly the acral type, can be challenging to treat due to the complex topography of the affected areas. In a case series by Goddard et al., six patients with eight acral CTCL lesions were treated with low-dose HDR-BT. This approach provided excellent local control, with rapid improvement and clinical clearance in all treated lesions. Only one lesion recurred locally during the 15.8-month follow-up period, and no long-term side effects were reported. Low-dose HDR-BT’s ability to provide homogeneous dosing over complex surfaces, such as the hands and feet, is a significant advantage in treating CTCL. The minimal acute toxicity observed in this study further supports the use of brachytherapy as a palliative treatment for CTCL, particularly in areas where EBRT may be less effective due to dose inhomogeneity [67].

For cutaneous lymphomas located in anatomically complex or sensitive areas, such as the face, custom-molded surface applicators can be used to deliver HDR-BT. A study by DeSimone et al. treated 23 facial lesions in 10 patients with mycosis fungoides, a type of CTCL. Using a surface applicator, patients received two fractions of 4 Gy each, and all patients completed the treatment successfully. The results showed a complete response in six patients and a partial response in the remaining four. Cosmetic outcomes were favorable, with mild erythema being the most common side effect. This technique allows for highly conformal dosing, ensuring that the radiation is delivered precisely to the lesion while sparing surrounding healthy tissue. This is particularly important for facial lesions, where cosmetic outcomes are a key consideration. The use of custom surface molds demonstrates the adaptability of brachytherapy in treating cutaneous lymphomas located in difficult-to-treat areas [68].

In a study by Devlin et al., six patients with eight lesions of cutaneous T-cell lymphoma on the hands and feet—a region particularly difficult for treatment due to cosmetic and functional concerns—were treated with high-dose-rate surface applicator brachytherapy. These areas present a challenge because their complex, curved surfaces make it difficult for other radiation techniques, like electron beams, to ensure even dose distribution [69].

The use of HDR brachytherapy allowed for precise control of the radiation dose, particularly by ensuring uniform coverage at a depth of 3 mm, the typical thickness of the lesions. The maximum surface dose was carefully managed to be less than 125% of the prescribed dose, which minimized hotspots and potential toxicity at the skin’s surface. Clinical outcomes were highly positive, with rapid improvement, complete clearance of all lesions, and minimal toxicity. No recurrences were observed during a follow-up period. This study highlighted the precision and effectiveness of brachytherapy for treating challenging CTCL cases on the hands and feet, warranting further investigation and validation across multiple institutions [69]. Shukla et al. presented another novel application of brachytherapy using the Freiburg Flap, a flexible mesh surface mold designed to ensure homogenous radiation coverage of curvilinear lesions. This method was used to treat a 64-year-old patient with MF, a type of CTCL, involving a large circumferential lesion on the forearm. Prior topical and phototherapy treatments had failed, prompting the consideration of radiation therapy. In this case, 16 catheters were placed in the Freiburg flap to deliver a total dose of 9 Gy over three fractions. The patient tolerated the treatment well, with only mild complications such as cellulitis and temporary radiation-induced neuropathy, both of which resolved without long-term effects. A complete response was achieved, and no recurrence was observed at nine months follow-up. This novel use of the Freiburg Flap demonstrated that brachytherapy could offer precise dose delivery even for complex and circumferential lesions, providing an excellent alternative to electron and photon beam therapy in difficult-to-treat locations [70].

High-dose rate brachytherapy has also been explored as an alternative treatment for FMF, particularly for lesions with complex topography. HDR brachytherapy provides effective target coverage while sparing normal tissue, resulting in fewer adverse effects and a shorter treatment course compared to electron beam radiotherapy [64].

Brachytherapy has proven to be an effective treatment modality for primary cutaneous lymphomas, offering high local control rates with minimal toxicity. Its ability to deliver precise radiation doses directly to the tumor site while sparing healthy tissue makes it an ideal choice for treating superficial lesions.

### 2.3. Skin Reactions During Radiotherapy

Radiotherapy is a cornerstone in the treatment of various malignancies, but it is often accompanied by a range of skin reactions that can significantly impact patient quality of life. These reactions, collectively termed radiation dermatitis, can manifest in various forms, including erythema, desquamation, and, in more severe cases, ulceration and necrosis. The incidence of skin reactions during radiotherapy is notably high, with estimates suggesting that approximately 85% to 95% of patients experience some degree of skin toxicity during their treatment [71,72,73,74]. Understanding the mechanisms, risk factors, and management strategies for these skin reactions is crucial for improving patient outcomes and comfort. The pathophysiology of radiation-induced skin reactions is primarily linked to the damage inflicted on the skin’s cellular structures by ionizing radiation. This damage leads to an inflammatory response characterized by erythema, which typically appears within the first two weeks of treatment [74]. The Radiation Therapy Oncology Group (RTOG) scale provides a standardized method for grading these skin reactions, which is essential for assessing treatment efficacy and planning supportive care. Understanding the RTOG grading system and its implications for patient management is crucial for healthcare providers involved in cancer care. The RTOG scale categorizes skin reactions into several grades, ranging from Grade 0 (no change) to Grade 4 (ulceration or necrosis). Grade 1 reactions include mild erythema, which may be accompanied by dryness or desquamation but does not involve moist desquamation. Grade 2 reactions are characterized by moderate erythema and dry desquamation, with less than 10% of the skin surface affected by moist desquamation. Grade 3 reactions involve marked erythema and moist desquamation, affecting more than 10% of the skin surface, while Grade 4 reactions indicate severe skin damage, including ulceration and necrosis [75,76]. The RTOG scale is presented in Table 2.

The RTOG grading system is particularly useful in clinical trials and practice as it allows for the standardization of toxicity assessments across different studies and treatment centers. For instance, in a study comparing conventional radiotherapy with hypofractionated radiotherapy, the RTOG scale was employed to evaluate acute skin toxicity, revealing that patients receiving hypofractionated regimens experienced lower rates of severe skin reactions compared to those receiving conventional treatment [75,79,80]. This highlights the importance of treatment planning and technique selection in minimizing skin toxicity. The severity of these reactions can vary based on several factors, including the total radiation dose, the fractionation schedule, and individual patient characteristics such as skin type and prior skin conditions [81,82]. For instance, patients with lighter skin tones may be more susceptible to severe reactions due to lower melanin content, which provides some degree of protection against radiation [81,83]. Acute skin reactions are generally classified into several categories, including mild erythema, dry desquamation, and moist desquamation. Mild erythema is often the first observable sign, typically occurring within 5 to 14 days post-irradiation [84]. As treatment progresses, patients may experience dry desquamation, characterized by flaking and peeling of the skin, which can be uncomfortable but is usually self-limiting. In contrast, moist desquamation, which involves the loss of the epidermis and exposure of the dermis, can lead to significant pain and increase the risk of infection [85]. Late skin reactions, such as fibrosis and telangiectasia, may develop months or even years after the completion of radiotherapy, further complicating patient management [86,87]. In addition to the RTOG grading system, various predictive factors have been identified that can influence the severity of radiation-induced skin reactions. These factors include the volume of skin irradiated, the use of bolus material, and concurrent chemotherapy.

For example, studies have shown that patients receiving concurrent chemotherapy are at a higher risk of developing severe radiation dermatitis due to the cumulative effects of both treatments on skin integrity [88]. Additionally, the location of the irradiated area plays a significant role; areas with skin folds or high moisture content, such as the axilla or groin, are more prone to severe reactions [81]. Management of radiation dermatitis involves a multifaceted approach aimed at prevention and treatment. Skin care regimens are critical and typically include gentle cleansing, moisturizing, and the use of barrier creams to protect the skin from further irritation [72,89]. The application of topical agents, such as corticosteroids or emollients, has been shown to alleviate symptoms and promote healing [90,91]. Furthermore, patient education on skin care practices is essential to empower patients to manage their skin health effectively during treatment [92,93]. Recent advancements in radiotherapy techniques, such as the use of hypofractionation and proton therapy, have shown promise in reducing the incidence and severity of skin reactions. These techniques allow for more precise targeting of tumors while sparing surrounding healthy tissue, thereby minimizing skin exposure to radiation [73,89,94]. To conclude, skin reactions during radiotherapy are a common and significant concern for patients undergoing cancer treatment. The high incidence of these reactions necessitates a comprehensive understanding of their underlying mechanisms, risk factors, and effective management strategies. Ongoing research into innovative radiotherapy techniques and supportive care interventions holds promise for improving patient outcomes and quality of life during and after treatment.

## 3. Discussion

PCLs represent a diverse group of lymphoproliferative disorders with varied biological behavior, ranging from indolent forms such as MF to more aggressive subtypes like cutaneous peripheral T-cell lymphoma (CPTCL) [95]. Due to the unique location and presentation of these malignancies, treatment strategies must be carefully tailored to balance efficacy with the preservation of quality of life, minimizing side effects and cosmetic damage. In this context, RT and BT have emerged as cornerstone modalities in the management of PCLs, offering targeted approaches for localized treatment.

Radiotherapy has long been recognized as an effective treatment for PCLs, especially for early-stage disease or localized lesions [96]. The precision of RT allows for targeted delivery of high doses of radiation to tumor sites while sparing surrounding healthy tissue. Studies have demonstrated favorable outcomes for patients with MF and other indolent forms, with response rates of up to 70–90% in early stages [16]. In cases of advanced or more aggressive PCLs, RT remains a viable option for symptom control and palliation, often in conjunction with systemic therapies [63].

Brachytherapy, a form of internal radiotherapy where a radiation source is placed close to the tumor, has recently gained attention in the treatment of cutaneous lymphomas. Its main advantage lies in its ability to provide highly localized radiation while minimizing the radiation dose to adjacent healthy skin and tissues. Several studies have reported favorable outcomes for brachytherapy in treating early-stage MF and other CTCLs, with high rates of complete and partial responses [67,68]. The main advantage of BT is its precision in delivering radiation to superficial cutaneous lesions, which are often difficult to treat with conventional external beam RT without affecting deeper structures. Modern advancements in BT, particularly through the application of 3D printing technology, demonstrate substantial improvements over traditional methods in the treatment of skin cancer [97]. By creating applicators based on CT imaging, 3D printing allows for precise customization to each patient’s unique anatomical features, resulting in superior dose conformity and fewer air gaps at the applicator–skin interface [98]. This individualized approach not only enhances radiation targeting but also minimizes exposure to surrounding healthy tissues, thereby reducing treatment-related side effects. The reported success of 3D-printed applicators in achieving complete remission in certain cases of inoperable skin cancer highlights the technology’s potential to improve patient outcomes significantly [99]. As this technology continues to show promise, further research and clinical trials are warranted to validate 3D printing as a standard practice in skin cancer brachytherapy. This advancement holds considerable promise for improving both the efficacy and safety of skin cancer treatments.

While both teleradiotherapy and BT have shown efficacy in treating primary cutaneous lymphomas, the choice between them depends on various factors, including the size, depth, and location of the lesion, as well as the patient’s overall clinical condition. External beam radiotherapy remains the gold standard for larger or deeper tumors, as well as for cases where brachytherapy may be technically challenging due to the anatomical location. However, for superficial, well-defined lesions, brachytherapy offers several advantages, including reduced skin toxicity and a potentially lower risk of long-term side effects like fibrosis and telangiectasia [15,100].

Recent comparative studies have explored the outcomes of teleradiotherapy versus BT in the management of PCLs, suggesting that while both modalities offer comparable long-term control, BT may provide superior cosmetic results due to its ability to deliver radiation more selectively to the tumor with minimal damage to surrounding tissues. Furthermore, the short treatment time and lower total radiation dose required for BT can be advantageous for patients, particularly those with more frail or elderly conditions [31]. Both teleradiotherapy and BT are associated with certain side effects, although these are generally manageable in the context of cutaneous lymphoma treatment. Acute skin reactions, including erythema, desquamation, and dermatitis, are common in both modalities but tend to be less severe with BT due to its more localized treatment approach [101]. Chronic side effects such as skin atrophy, fibrosis, and pigment changes may occur, particularly in cases of long-term teleradiotherapy use. BT’s major advantage in terms of side effect profile is its lower incidence of these long-term sequelae, which is critical for patients with PCLs who often require repeated courses of therapy over many years. Additionally, due to its ability to deliver radiation precisely to the tumor with minimal damage to surrounding healthy skin, BT is less likely to cause the more severe complications seen with traditional external teleradiotherapy, such as radiation-induced skin ulcers or second malignancies [102]. Despite the promising results associated with both RT techniques, certain limitations exist. One major limitation is the difficulty in treating larger or more advanced PCL lesions using brachytherapy due to the technical constraints of the therapy and the risk of underdosing deeper tissues. Additionally, BT requires specialized equipment and expertise, which may not be widely available in all centers [102]. Teleradiotherapy, while more widely accessible, can lead to more significant long-term skin damage and requires careful patient management to mitigate side effects, especially in cases of repeated or long-term treatments. The long-term risk of secondary malignancies, particularly in younger patients, remains a concern, although these risks are relatively low compared to more aggressive treatment modalities like chemotherapy [103].

Despite the clear efficacy of radiotherapy in primary cutaneous lymphomas, several real-world limitations remain that may influence its broader application. Access to advanced techniques such as high-dose-rate brachytherapy (HDR-BT) or total skin electron beam therapy (TSEBT) can be restricted to specialized centers, limiting availability in many regions, particularly in low- and middle-income countries. Even where equipment is available, the high cost of installation, maintenance, and specialized expertise may pose barriers to routine use, raising concerns about cost-effectiveness compared to other local or systemic therapies. Moreover, variability in practice patterns across institutions highlights a lack of standardized protocols, making it difficult to generalize outcomes globally. These challenges underscore the need for further health-economic analyses, development of simplified and resource-adapted treatment approaches, and international collaboration to ensure equitable access to effective radiotherapy for patients with primary cutaneous lymphomas.

Future research in the treatment of PCLs with teleradiotherapy and BT should focus on optimizing radiation doses, refining patient selection criteria, and improving the combination of these modalities with novel systemic therapies. Advances in imaging technology and radiation planning can further enhance the precision of both external beam radiotherapy and brachytherapy, improving outcomes and minimizing toxicity. Moreover, further studies are needed to explore the optimal role of BT in the treatment of advanced or refractory PCLs. Given the promising results for early-stage disease, future trials may investigate the use of BT in combination with systemic therapies to improve outcomes in more aggressive forms of cutaneous lymphomas.

## 4. Conclusions

In conclusion, teleradiotherapy and brachytherapy remain highly effective and well-established modalities in the treatment of primary cutaneous lymphomas (PCL), providing targeted control with favorable safety and cosmetic outcomes. Teleradiotherapy continues to be the standard for larger or deeper lesions, while brachytherapy has shown particular value for superficial, localized disease where precision and tissue preservation are paramount. Recent advances in radiotherapy biology highlight not only the direct cytotoxic effects of ionizing radiation but also its ability to modulate the tumor microenvironment and enhance antitumor immune responses, offering new opportunities for integration with systemic therapies. Ongoing clinical studies, such as trials combining low-dose total skin electron beam therapy (TSEBT) with agents including mogamulizumab (NCT04128072), brentuximab vedotin (NCT02822586), or mechlorethamine gel [104], underscore the potential of combining radiotherapy with novel targeted and immunotherapeutic approaches. Future strategies should focus on refining dose and fractionation to balance efficacy with toxicity and on leveraging the immunomodulatory properties of radiotherapy to achieve durable systemic disease control. Multidisciplinary collaboration between dermatologists, hematologists, and radiation oncologists will be crucial to translating these evolving strategies into improved outcomes and quality of life for patients with PCL.

## Figures and Tables

**Table 2:** Radiation Therapy Oncology Group (RTOG) acute toxicity scoring system, skin [76,77,78].

Grade	Observation
Grade 0	None
Grade 1	Follicular, faint, or dull erythema, epilation, dry desquamation, decreased sweating. Mild tightness of skin and itching may occur.
Grade 2	Brisk erythema/dry desquamation. Skin may feel tight, sore and itchy.
Grade 2.5	Patchy moist desquamation. Yellow/pale green exudate may be visible on surface. Soreness and oedema.
Grade 3	Confluent, moist desquamation other than skin folds, pitting edema. Yellow/pale green exudate visible. Soreness. Bleeding may occur.
Grade 4	Ulceration, necrosis, hemorrhage

## Data Availability

Data are available on request from the corresponding author.
